# Social-psychological determinants of hormonal contraceptive use intentions among adolescent girls in the Bono East Region of Ghana

**DOI:** 10.3389/fpubh.2023.1110112

**Published:** 2023-08-01

**Authors:** Ellen Abrafi Boamah-Kaali, Robert A. C. Ruiter, Seth Owusu-Agyei, Kwaku Poku Asante, Fraukje E. F. Mevissen

**Affiliations:** ^1^Kintampo Health Research Centre, Research and Development Division, Ghana Health Service, Kintampo, Ghana; ^2^Faculty of Psychology and Neuroscience, Maastricht University, Maastricht, Netherlands; ^3^Institute of Health Research, University of Health and Allied Sciences, Ho, Ghana; ^4^Department of Public Health, Municipal Public Health Service Rotterdam-Rijnmond, Rotterdam, Netherlands

**Keywords:** adolescent women, hormonal contraceptive, social-psychological, determinants, attitudes, self-efficacy, Ghana

## Abstract

**Introduction:**

The correct and consistent use of hormonal contraceptive (HC) methods by sexually active adolescent girls can prevent pregnancy and avert the health and social consequences of unwanted pregnancy for both the mother and her child. Despite these benefits, research shows that HC use is rather low among adolescent girls globally and especially among those in low and middle-income countries. This study was carried out to assess the social-psychological determinants of HC use intentions among adolescent girls and young women.

**Methods:**

A cross-sectional survey was conducted among 1,203 young women aged 15–24 years from 70 communities within the Kintampo North Municipality and Kintampo South District in the Bono-East Region of Ghana from April 2021 to September 2021. Multiple linear regression analysis was used to identify factors associated with the intention to use HC among the entire sample of 1,203 respondents and among two sub-samples of young women based on HC use experience.

**Results:**

Attitude toward personal HC use (β = 0.268; *p* < 0.001), self-efficacy toward access and use of HC (β = 0.341; *p* < 0.001), and HC use experience (β = 0.647; *p* < 0.001) were found to be significant and unique correlates of HC use intention among the entire sample of adolescent girls. Attitude toward personal HC use and self-efficacy toward access and use of HC were also associated with HC use intention in the two sub samples significantly (*p*’s < 0.001). In addition, among participants with no HC experience, being a Christian as opposed to participants that affiliate themselves with Islam, Traditional religion or being non-religious positively predicts future HC use (β = 0.230; *p* < 0.01).

**Conclusion:**

Our results demonstrate that different groups of adolescent girls need different interventions, focusing on different determinants for the motivation to use HC. Comprehensive sexuality education, informing all adolescent girls about the personal benefits of HC use and enhancing their skills in accessing and using HCs, can support their HC use intentions to promote their reproductive health and general wellbeing.

## Introduction

Worldwide, an estimated 21 million adolescent girls get pregnant every year, contributing significantly to maternal morbidities and mortalities (1–4). Socially, young mothers who experience unplanned and unwanted pregnancies are stigmatized; many are unable to complete their education and are not able to secure good jobs (5, 6). Adolescent girls are more likely to abort their unwanted pregnancies using unsafe methods that endanger their lives and affect their future reproductive health and general well-being (7). Neonates born to adolescent mothers have a higher risk of low birth weight, preterm delivery and poor neonatal outcomes (8, 9). They may also suffer physical and psychological abuses and become poor achievers in the future (4, 10).

The correct and consistent use of hormonal contraceptives (HC) by sexually active adolescent girls can prevent pregnancy and avert the health and social consequences for the mother, her child, and the family (11, 12). Also, women who use HC are empowered to make decisions regarding their reproductive health and overall wellbeing (7) In addition, the use of HC promotes sustainable population growth in less developed countries, ensuring equitable distribution of resources and poverty reduction (13, 14).

Despite these benefits, research shows that effective use of HC is rather low among sexually active adolescent girls globally with Ghana as no exception (15–18). In 2019, 43% of the 32 million adolescent girls in Low-and-Middle-Income Countries who wanted to prevent pregnancy at least in the next two years were not using any method of contraception (12). In Ghana, as much as 51% of sexually active adolescent girls who did not want to get pregnant in 2017 were likewise not using any family planning method (19). Additionally, adolescents more often use condoms (20–22) compared to HC because of their ready availability and dual protective property (23). However, condoms are less effective at preventing pregnancy compared to hormonal methods (24). Moreover, high dependence on partner support is required but many adolescent girls, especially in sub-Saharan Africa, are less autonomous and struggle to negotiate for condom use (15, 24–26). Further, consistent condom use is highly reduced over time among people in stable relationships (23). Therefore, relying on condoms only for pregnancy prevention because of their dual protective option is not a comprehensive approach. The double Dutch approach (i.e., using condom in addition to a hormonal contraceptive method) seem the best approach for preventing pregnancy and disease simultaneously.

Interventions toward the double Dutch approach demand a needs assessment to understand all the factors that influence the use of condoms and HCs. Research on determinants of condom use is widely reported in the literature (25, 27–31). However, data on the determinants of adolescent HC use is limited. This emphasizes the need to understand why sexually active adolescent girls do not use HCs. Our study aimed to understand the socio-psychological determinants of HC use intentions among adolescent girls as evidence for future interventions toward improved HC use and simultaneous use of HCs and condoms when needed.

Previous research on the socio-psychological determinants of hormonal contraceptive use among adolescents reports that attitudes to HC, normative beliefs on HC use, perceived partner, peer and parental norms regarding HC use, perceived susceptibility to pregnancy, perceived risk of pregnancy complications, and perceived benefits of HC use, influence HC use among adolescents in LMIC (15, 16, 32–42). Additionally, barriers to HC use, including perceived (e.g., future infertility, epilepsy) and real HC side effects (including weight gain, amenorrhea, decreased libido, headaches, depression, irregular menses, heavy menses, brain changes) negatively affect HC use among adolescent girls (43). Moreover, lack of confidence to successfully discuss contraceptive use with the partner, to ask for contraceptives from a health facility, and use contraceptives have been found to influence contraceptive use (33, 34, 41, 44, 45).

In addition to empirical evidence, theories can further explain what factors may influence HC use (46). Two theories that may provide further insight are the Theory of Planned Behavior (TPB) and the Health Belief Model (HBM). The TPB postulates that the intention to perform any given behavior precedes its actual performance provided people have the necessary skills and barriers do not hinder the behavioral execution process. Intention in turn is influenced by attitudes, subjective norms, and perceived behavioral control (47, 48). That is, applied to hormonal contraception use, adolescent girls’ intentions are higher when they are favorably disposed to hormonal contraceptive use (attitude), perceive that important people around them approve their use HC (subjective norm), and have the confidence to use HC themselves (self-efficacy) and perceive sufficient autonomy to make their personal decision (perceived behavioral control).

The HBM, in turn, argues that the decision to perform a given health-related behavior is motivated by evaluations of perceived risk and the perceived costs and benefits of recommended self-protective action (49). When applying HBM to our study, adolescents will consider using HC if they consider themselves to be vulnerable to unwanted pregnancy and if they perceive the outcome of unwanted pregnancy to be severe, while acknowledging that using HC benefits them and they anticipate no barriers to using HCs.

Previous research in Kintampo, Ghana, has studied the individual and environmental factors of HC use, using qualitative research methods (50). Results from these studies demonstrate the important role of social-psychological constructs in explaining intention and behavior toward HC use. Especially, positive attitudes toward HC use and self-efficacy in organizing and use of HC were found to motivate the uptake and consistent HC use. However, fear of the side effects of HC and strong cultural and religious norms against teenage sexuality and HC use seem to negatively influence (consistent) HC use. Although these qualitative studies have identified potential psychosocial determinants of adolescent decision-making concerning correct and consistent hormonal contraceptive use, they do not provide us with correlational evidence and they are not able to provide estimates of the relative importance of these determinants in predicting the motivation to start HC use. Therefore, the relative significance of the different factors predicting HC use are still unknown.

This current study aimed to assess the relative importance of earlier identified social cognitive constructs in the prediction of HC use intentions among adolescents as evidence for informing effective hormonal contraceptive use interventions. Unlike social demographic and personality-related variables, social cognitive variables are amenable to change because they are based on beliefs people hold. These beliefs can be targeted with theory-and evidence-based educational interventions providing information and training through a multitude of behavioral change methods including persuasion, modeling, and guided practice (46).

## Methods

### Study design

A cross-sectional survey was conducted among 1,203 young women in 70 communities within the Kintampo North Municipality and Kintampo South District in the Bono-East Region of Ghana from April 2021 to September 2021.

### Study area description

The Kintampo North Municipality and Kintampo South District are part of a Health and Demographic Surveillance System (KHDSS) carried out as a core activity of the Kintampo Health Research Centre (KHRC). The KHDSS area has a current population of 173,487 of which the young adult population (15 years to 24 years) is 32,984 (49.8% females and 50.2% males). Study participants for the study were recruited from the catchment area of the KHDSS.

### Participant identification, recruitment, and consenting

Participants for this study were identified through household surveillance by field staff of the Kintampo Health Research Centre who are based in the KHDSS communities. During the surveillance, any female between 15 to 24 years of age who they met was invited to participate in our study that explored determinants of hormonal contraceptive use. There were no exclusion criteria besides the stated age limits.

All prospective study participants received detailed information on the study and their rights as study participants. They were informed that participation was voluntary, which meant they could opt out of the study at any time without offering any explanations and they could refuse answering questions they did not want to answer. They were allowed to ask questions concerning their rights as study participants which were duly answered. Participants’ names were not written instead they were given unique study ids for the sake of anonymity. They were assured of private storage of their data on highly secured computers protected from non-study staff access with passwords. All study participants provided written informed consent for participating in the study. Participants who were less than 18 years of age had to provide assent and their parents or legally authorized representatives consented to the study before they could participate. Based on their preference, study participants could opt to have the interview right away after consenting or could schedule to be interviewed later at a preferred location.

### Sample description

A total of 1,203 young women aged 15 to 24 years with a mean age of 20.4 years (SD = 1.84) participated in the study. Referring to educational achievement, 52.5%% had primary or Junior High School education, 37.6% had a Senior High School or tertiary education and 10.7% had never been to school. Forty percent (45%) lived with two parents, 28.4% had other living arrangements, and 26.6% lived with one parent. Regarding religion, 67.2% of them were affiliated to Christianity, 29% were Muslims, and 3.8%. were either Traditional worshippers or were without religion. The majority (39.1%) of the respondents were students, 25.9% of them were in a vocational training, 21.4% were unemployed, and 13% had a job. Most (39.9%) respondents were single, 33.2% were in a steady relationship, 13.7% were in a casual relationship and 13.2% were married. As much as 62.7% (814/1203) were sexually active, and 57.2% (n = 466/814) of those had experienced pregnancy. Hormonal contraceptive use was reported among 29.1% of respondents and 70.9% had no experience with HC. See Table 1 for a detailed description of the sample.

**Table 1:** Sample description (*N* = 1,203).

Sociodemographic variables	Frequency (%)
Level of education	
Primary/Junior High	632 (52.5%)
SHS/Tertiary	442 (37.6%)
No education	129 (10.7%)
Living arrangement	
Two parents	541 (45%)
Other living arrangements	342 (28.4%)
One parent	320 (26.6%)
Religion	
Christianity	808 (67.2%)
Islam	349 (29%)
Traditional / No religion	46 (3.8%)
Current occupation	
Student	470 (39.1%)
Vocational training	312 (25.9%)
Unemployed	257 (21.4%)
In a job	164 (13.6%)
Relationship status	
Single	480 (39.9%)
Steady relationship	399 (33.2%)
Casual relationship	165 (13.7%)
Married	159 (13.2%)
Sex experience	
Yes	814 (62.7%)
No	389 (37.3%)
Do not want to answer	
* Pregnancy experience (n = 814)	
Yes	466 (57.2%)
No	339 (41.6)
Do not want to answer	9 (1.1%)
Hormonal contraceptive experience	
Yes	350 (29.1%)
No	853 (70.9%)

* Among only adolescent girls and young women with sexual experience.

### Data collection

Data was collected electronically with tablets using the Open Data Kit (ODK) software. Trained field workers administered the questionnaire to study participants using face-to-face interviews either in Twi (local language) or English version of the questionnaire based on participant’s preference.

### The questionnaire

The selection of determinants to be included in the questionnaire was based on the empirical literature, health behavior theories and results of three earlier qualitative studies in the study area that explored the individual and environmental determinants of uptake and the correct and consistent use of HCs among adolescent girls (31, 39). The formulation of items was guided by the UNDP/UNFPA/WHO/World Bank illustrative questionnaire for interview surveys with young people (51) and the survey construction guidelines of Fishbein and Ajzen (52). The questionnaire was developed in English Language and was translated into Twi by a research officer experienced in translation from English to Twi. Back-translation of the Twi version into English was done to check for consistency in content and meaning by another research officer, skilled in translation from Twi to English. The Twi version of the questionnaire was reviewed and finalized by a research fellow with a master’s degree in Public Health who is proficient in English and Twi. Both Twi and English versions of the questionnaire were used for data collection.

The questionnaire first measured the sociodemographic characteristics of the participants including age, level of education (responses: no education, primary, JHS, SHS, tertiary), living arrangements (responses: mother, father, both parents, other relatives, on my own, other living arrangements), religion (responses: Christianity, Islam, Traditional religion, no religion), current occupation (responses: student, learning a trade/vocation, teaching, nurse, clerical work, trading, none) and relationship status (responses: married, serious relationship, casual relationship, single, divorced, widowed). This was followed by a range of 5-point Likert scales measuring psychosocial determinants of HC use, including attitude toward HC use (sub-sections: Attitude toward HC product, attitude toward HC use among adolescents, attitude toward personal HC use, attitude toward discussing HC use), future ambition, subjective norms (sub-sections: Partner HC norm, mother’s HC norm, peer HC norm), perceived behavioral control (sub-sections: Self-efficacy, self-regulation skills), sexual morality, compliance, risk perception toward getting pregnant as an adolescent, severity of consequences of getting pregnant as an adolescent, sexual and contraceptive experience (sub-sections: discussing SRH issues with mother/ caretaker, relationship experience, sexual intercourse experience, pregnancy experience) and behavioral intention toward HC use.

### Data analysis

Data was exported from ODK to Excel and transferred to SPSS for analysis. The analysis was carried out with SPSS version 27 IBM. We used factor and reliability analyses to support our decision on which items could be combined into scales measuring the same psychosocial construct. A mean score was calculated for items that belonged to the same psychosocial construct. Internal consistency between items was considered sufficient with a Cronbach’s alpha of α > 0.60 or Pearson correlation coefficient *r* > 0.50. See Table 2 for all social-psychological constructs measured, the number of items per construct, example items and answer scales used, and corresponding internal validities. We plotted histograms to observe normality of the data by observing skewness and kurtosis for all the social psychological variables measured. No extreme values were observed ({skewness scores < −0.03}; {Kurtosis >2.6}).

**Table 2:** Social-psychological variables and their internal validities.

Variables	Number of items	Cronbach’s alpha (α)/Pearson r	Example questions
Intention to use HC 1 = low−5 = high	3	α = 0.82	I intent to use hormonal contraceptives within the upcoming 3 months 1 = strongly disagree–5 = strongly agree
Attitude toward HC product 1 = negative – 5 = positive	3	α = 0.91	Using hormonal contraceptives to protect against unwanted pregnancy seems to me 1 = very harmful–5 = very beneficial
Attitude toward adolescent HC use 1 = negative – 5 = positive	6	α = 0.92	Using hormonal contraceptives as an adolescent seems to me 1 = very bad–5 = very good
Attitude toward personal HC use 1 = negative – 5 = positive	5	α = 0.81	For me, when I am in a serious sexual relationship it makes sense to always use hormonal contraceptives
Attitude toward discussing HC use with mother 1 = negative – 5 = positive	3	α = 0.94	To discuss hormonal contraceptive use with my mum/care taker seems to me 1 very foolish–5 = very wise
Attitude toward discussing HC use with partner 1 = negative – 5 = positive	3	α = 0.95	Discussing hormonal contraceptives with my partner seems to me 1 = very unimportant – 5 = very important
Attitude toward discussing HC use with friends 1 = negative – 5 = positive	3	α = 0.93	Discussing hormonal contraceptives with my friends seems to me 0.1 = very unimportant–5 = very important
Self-efficacy toward access and use of HC 1 = low−5 = high	6	α = 0.88	I am confident that I can go to the drugstore/pharmacy myself to get a hormonal contraceptive, even if the pharmacist is going to ask me questions. 1 = strongly disagree–5 = strongly agree
Self-efficacy to discuss HC use with important others 1 = low−5 = high	3	α = 0.77	I am confident that I can discuss hormonal contraceptive use with my mother/caretaker. 1 strongly disagree–5 = strongly agree
Self-regulation skills 1 = low−5 = high	3	α = 0.71	I am confident that I can continue to use hormonal contraceptives, even if I have to secretly use it. 1 = strongly disagree – 5 = strongly agree
Mother’s normative belief 1 = negative – 5 = positive	3	α = 0.86	My mother/caretaker thinks that hormonal contraceptives are good for teenage girls to use. 1 = strongly disagree – 5 = strongly agree
Friend’s normative belief 1 = negative – 5 = positive	3	α = 0.84	My friends will/approve for me to use hormonal contraceptives. 1 = strongly disagree – 5 = strongly agree
Partner normative belief 1 = negative – 5 = positive	3	α = 0.84	My boyfriend thinks that hormonal contraceptives are good for teenage girls to use. 1 = strongly disagree – 5 = strongly agree
Discussing SRH issues with mother 1 = low−3 = high	3	α = 0.88	How often do you talk with your mother/caretaker about hormonal contraception? 1 = never–3 = often
Risk perception toward getting pregnant 1 = low−5 = high	3	α = 0.63	The likelihood of me getting pregnant when I am having sex without using hormonal contraceptives is…1 = very unlikely to 5 = very likely
Severity of consequences of getting pregnant as an adolescent 1 = low−5 = high	3	α = 0.88	The health consequences of getting pregnant as a teenage girl are severe. 1 = strongly disagree–5 = strongly agree
Future ambition 1 = low–5 = high	3	α = 0.75	I enjoy making plans for the future and making them a reality. 1 = strongly disagree–5 = strongly agree
Sexual morality 1 = low −5 = high	2	r = −0.03	Using hormonal contraceptives promotes promiscuity among teenagers. 1 = strongly disagree–5 = strongly agree

Additionally, we dummy coded the demographic measures. Level of education which originally had five categories resulted in three categories including (no education, primary/JHS, SHS/tertiary). We combined respondents with primary and JHS levels of education into “primary/JHS” and those with SHS and tertiary levels into “SHS/tertiary,” while maintaining the “no education” category and considering that as the reference category in the regression analyses. Living arrangements, which originally had six categories resulted in three namely (one parent, two parents, other living arrangements). Participants living with either mother or father were combined into one category as living with “one parent.” Participants living with both parents were classified as living with “two parents,” those living on their own, or living with other relatives or having other living arrangements were combined into the “other living arrangement” category, and considered the reference category. In addition, religion which originally had four categories resulted in three as (Christianity, Islam, Traditional/no religion). Those affiliated to the Islamic or Traditional religions and those with no religion were combined into the “Islam/Traditional/no religion” category and used as the reference group for the regression analyses. Further, current occupation with previous seven categories resulted in four categories as (student, vocational training, in a job, unemployed). Teachers, nurses, clerical workers and those trading were put together and categorized as “in a job.” Those in the “none” category was recoded as the “unemployed” category and considered the reference category. Lastly, relationship status with original six categories produced four categories called (married, single, steady relationship, casual relationship). Respondents who were widowed, divorced or single were combined into the “single category” and used as the reference category.

We performed descriptive analyses for all background variables reporting frequencies and percentages for each category. We tested the correlation between all social-psychological variables and HC use intention. Measures that showed significant univariate associations with intention at a significance level of *p* < 0.01 were included in a stepwise multiple linear regression analysis. In the first step, we entered the social-psychological variables (enter method). Next, HC use experience was added to control its effect on the variables in step one. In the third step, the socio-demographic variables were added to correct for the influence of demographic variations on the variables that appeared significant in step one and two. The variable “HC use experience” seemed to correlate well with intention in the second and final regression models. We therefore repeated the correlation and regression analyses for participants with HC use experience (past and current use) and those without (never used HC).

### Ethical approval

We received ethical approvals for the conduct of this research from the Kintampo Health Research Centre Institutional Ethics Committee (Federal Wide Assurance number 00011103) in Ghana and the Ethical Review Committee, Psychology, and Neuroscience at Maastricht University (ECP_04_09_2012_S23) in the Netherlands.

## Results

### Correlations for the entire sample (*N* = 1,203)

Table 3 provides the association strengths (Pearson correlation *r*) between all social-psychological variables and hormonal contraceptive use intentions for the entire sample of adolescent girls and young women. According to Cohen (53), correlations of *r* = 0.10 to 0.23 indicate a small effect size; correlations of *r* = 0.24 to 0.36 show a moderate effect size, and large effect sizes have a correlation of *r* ≥ 0.37 (53). All reported associations showed a positive correlation with intention, with higher scores on the indicated measure resulting in a more positive intention (*p*’s < 0.05). As shown in Table 3, attitude toward adolescent HC use, attitude toward personal HC use, self-efficacy toward access and use of HC, and self-regulation skills showed large positive associations with intention to use HC among all adolescent girls and young women (*r*’s > 0.37, *p*’s < 0.01). Attitude toward HC product, self-efficacy to discuss HC use with important others, mothers’, friends’ and partners’ normative belief toward HC use were each positively but moderately associated with intention to use HC among all adolescent girls and young women (*r’s* > 0.24, *p*’s < 0.01). Also, attitude toward discussing HC use with mother, friends and partner, discussing of SRH issues with mother, pregnancy risk perception, future ambition, and HC knowledge were positively associated with HC use intentions but showed weak associations with intention to use HC. Likewise, the perceived severity of pregnancy consequences showed very weak association with HC use intention (*r*’s < 0.23, *p*’s < 0.01). Sexual morality and age were not significantly associated with intention to use HC (*p*’s > 0.05) and were not included in the regression model.

**Table 3:** Correlation analysis of social psychological variables and HC use intentions among entire sample of adolescent girls and young women (*N* = 1,203).

	Mean (SD)	1	2	3	4	5	6	7	8	9	10	11	12	13	14	15	16	17	18	19	20
Intention to use HC	2.78 (1.13)																				
Attitude toward HC product	3.38 (1.10)	0.366^**^																			
Attitude toward adolescent HC use	3.31 (1.01)	0.387^**^	0.904^**^																		
Attitude toward personal HC use	3.27 (0.91)	0.490^**^	0.548^**^	0.572^**^																	
Attitude toward discussing HC use with mother	3.35 (1.08)	0.225^**^	0.361^**^	0.369^**^	0.347^**^																
Attitude toward discussing HC use with friends	3.45 (1.07)	0.222^**^	0.414^**^	0.451^**^	0.440^**^	0.459^**^															
Attitude toward discussing HC use with partner	3.70 (1.05)	0.197^**^	0.295^**^	0.343^**^	0.324^**^	0.364^**^	0.414^**^														
Self- efficacy toward access and use of HC	3.38 (0.93)	0.527^**^	0.468^**^	0.470^**^	0.554^**^	0.295^**^	0.335^**^	0.352^**^													
Self -efficacy to discuss HC use with important others	3.44 (0.93)	0.288^**^	0.348^**^	0.358^**^	0.372^**^	0.510^**^	0.459^**^	0.435^**^	0.497^**^												
Self -regulation skills	3.00(0.94)	0.431^**^	0.426^**^	0.449^**^	0.476^**^	0.336^**^	0.326^**^	0.241^**^	0.556^**^	0.438^**^											
Mother’s normative belief on HC	3.17 (0.99)	0.293^**^	0.442^**^	0.459^**^	0.388^**^	0.482^**^	0.395^**^	0.334^**^	0.395^**^	0.462^**^	0.428^**^										
Friend’s normative belief on HC	3.28 (0.95)	0.277^**^	0.432^**^	0.476^**^	0.396^**^	0.326^**^	0.522^**^	0.350^**^	0.426^**^	0.469^**^	0.427^**^	0.644^**^									
Partner normative belief on HC	3.30 (1.00)	0.254^**^	0.319^**^	0.405^**^	0.382^**^	0.252^**^	0.363^**^	0.547^**^	0.374^**^	0.357^**^	0.287^**^	0.551^**^	0.522^**^								
Discussing SRC issues with mother	1.42 (0.50)	0.197^**^	0.138^**^	0.124^**^	0.165^**^	0.302^**^	0.141^**^	0.159^**^	0.188^**^	0.244^**^	0.237^**^	0.266^**^	0.124^**^	0.151^**^							
Perceived severity of consequences of Pregnancy	3.80 (0.92)	0.078^**^	0.056	0.007	0.111^**^	0.033	0.037	0.006	0.101^**^	0.044	0.107^**^	0.042	0.024	0.036	0.028						
Pregnancy risk perception	3.55 (0.90)	0.210^**^	0.206^**^	0.190^**^	0.265^**^	0.153^**^	0.178^**^	0.279^**^	0.298^**^	0.300^**^	0.266^**^	0.231^**^	0.254^**^	0.238^**^	0.105^**^	0.274^**^					
Future ambition	4.00 (0.76)	0.124^**^	0.169^**^	0.187^**^	0.285^**^	0.172^**^	0.234^**^	0.281^**^	0.242^**^	0.165^**^	0.145^**^	0.195^**^	0.179^**^	0.342^**^	0.020	0.301^**^	0.169^**^				
Sexual morality	3.31 (1.04)	−0.033	−0.088^**^	−0.082^**^	0.043	0.051	0.007	0.011	0.022	0.034	0.096^**^	0.012	−0.014	0.023	0.024	0.177^**^	0.059^*^	0.137^**^			
HC knowledge	3.02 (1.25)	0.165^**^	0.282^**^	0.300^**^	0.187^**^	0.103^**^	0.092^**^	0.115^**^	0.247^**^	0.106^**^	0.175^**^	0.184^**^	0.177^**^	0.174^**^	0.079^**^	−0.077^**^	0.056	0.028	−0.029		
Age	20.40 (1.84)	0.017	0.134^**^	0.115^**^	0.050	0.060^*^	0.086^**^	0.103^**^	0.128^**^	0.159^**^	0.065^*^	0.076^**^	0.083^**^	0.078^*^	0.100^**^	−0.081^**^	0.108^**^	−0.015	−0.020	0.155^**^	

** . Correlation is significant at the 0.01 level (2-tailed).

### Multivariable linear regression analysis of intention to use HC among the entire sample (*N* = 1,203)

Together the significant univariate psychosocial correlates of intention explained 36% of the variance in intention, with significant unique associations for attitude toward personal HC use, self-efficacy toward access and use of HC, and self-regulation skills at the *p* < 0.01 level. Adding the behavioral variable HC experience to the regression model significantly improved the explained variance in intention to 42.1%, *Fchange* (1, 704) = 73.88, *p* < 0.001. Adding the demographic variables, resulted in a total explained variance of 43.6% in intention toward HC use, *Fchange* (13, 691) = 1.40, *p* = 0.15. In the final regression model, the attitude toward personal HC use, self-efficacy toward access and use of HC and previous HC use, remained significant, but self-regulation skills, which was significant in step 1, lost its significance in steps 2 and 3 at *p* < 0.01 All other measures did not show significant unique associations with intention (*p*’s > 0.01) (see Table 4).

**Table 4:** Multivariable linear regression analysis of intention to use HC among entire sample of adolescent girls and young women (*N* = 1,203).

	Step 1: multivariable linear regression analysis of HC use intentions among entire adolescent girls and young women			Step 2: controlling for HC use experience			Step 3: demographic variables included		
	Unstandardized coefficients (SE)	*p*-value	95% C.I (Lower, Upper bound)	Unstandardized coefficient (SE)	*p*-value	95% C.I (Lower bound, Upper bound)	Unstandardized Coefficient (SE)	*p*-value	95.0% C. I (Upper, Lower Bound)
(Constant)	0.006 (0.257)	0.982	(−0.500, 0.511)	0.284 (0.247)	0.250	(−0.201, 0.770)	0.249 (0.274)	0.366	(−0.290, 0.787)
Attitude toward HC product	−0.054 (0.075)	0.475	(−0.201, 0.094)	−0.037 (0.072)	0.608	(−0.177, 0.104)	−0.031 (0.072)	0.664	(−0.172, 0.109)
Attitude toward adolescent HC use	0.137 (0.086)	0.110	(−0.031, 0.305)	0.086 (0.082)	0.291	(−0.074, 0.247)	0.095 (0.082)	0.248	(−0.066, 0.255)
Attitude toward personal HC use	0.302 (0.052)	0.000	(0.200, 0.404)	0.285 (0.049)	0.000	(0.188, 0.382)	0.268 (0.050)	0.000	(0.170, 0.365)
Attitude toward discussing HC use with mother	0.004 (0.042)	0.926	(−0.078, 0.086)	−0.019 (0.040)	0.633	(−0.098, 0.059)	−0.024 (0.040)	0.553	(−0.102, 0.055)
Attitude toward discussing HC use with friends	−0.048 (0.043)	0.265	(−0.132, 0.036)	−0.038 (0.041)	0.349	(−0.118, 0.042)	−0.036 (0.041)	0.377	(−0.116, 0.044)
Attitude toward discussing HC use with partner	−0.038 (0.043)	0.377	(−0.122, 0.046)	−0.033 (0.041)	0.420	(−0.113, 0.047)	−0.029 (0.041)	0.476	(−0.110, 0.051)
Self-efficacy to access and use HC	0.381 (0.052)	0.000	(0.280, 0.482)	0.354 (0.049)	0.000	(0.258, 0.451)	0.341 (0.050)	0.000	(0.244, 0.438)
Self-efficacy to discuss HC use with important others	−0.031 (0.051)	0.536	(−0.131, 0.068)	−0.010 (0.048)	0.834	(−0.105, 0.085)	−0.007 (0.048)	0.880	(−0.102, 0.088)
Self-regulation skills	0.142 (0.048)	0.003	(0.047, 0.236)	0.104 (0.046)	0.024	(0.014, 0.194)	0.095 (0.046)	0.038	(0.005, 0.186)
Mother’s normative belief on HC	0.018 (0.053)	0.735	(−0.086, 0.122)	0.009 (0.050)	0.859	(−0.090, 0.108)	0.008 (0.051)	0.874	(−0.091, 0.107)
Friend’s normative belief on HC	−0.026 (0.054)	0.623	(−0.132, 0.079)	−0.031 (0.051)	0.547	(−0.132, 0.070)	−0.023 (0.051)	0.657	(−0.124, 0.078)
Partner normative belief on HC	0.024 (0.050)	0.629	(−0.073, 0.121)	0.023 (0.047)	0.632	(−0.070, 0.115)	0.022 (0.048)	0.651	(−0.072, 0.116)
Discussing SRH issues with mother	0.161 (0.074)	0.029	(0.016, 0.305)	0.091 (0.071)	0.198	(−0.048, 0.229)	0.105 (0.071)	0.139	(−0.034, 0.244)
Severity of consequences of pregnancy	0.019 (0.041)	0.642	(−0.061, 0.099)	0.021 (0.039)	0.588	(−0.055, 0.097)	0.029 (0.039)	0.459	(−0.048, 0.106)
Pregnancy risk perception	0.032 (0.043)	0.449	(−0.051, 0.116)	0.029 (0.041)	0.481	(−0.051, 0.108)	0.038 (0.041)	0.356	(−0.042, 0.118)
Future ambition	−0.067 (0.051)	0.190	(−0.168, 0.033)	−0.065 (0.049)	0.183	(−0.161, 0.031)	−0.062 (0.049)	0.204	(−0.158, 0.034)
HC knowledge	0.002 (0.029)	0.948	(−0.055, 0.059)	0.000 (0.028)	0.986	(−0.054, 0.055)	−0.010 (0.028)	0.727	(−0.065, 0.045)
HC experience				0.659 (0.077)	0.000	(0.508, 0.810)	0.647 (0.080)	0.000	(0.490, 0.805)
Ever been pregnant							0.017 (0.079)	0.834	(−0.138, 0.171)
Primary/JHS							−0.052 (0.113)	0.645	(−0.274, 0.170)
SHS/tertiary							−0.007 (0.126)	0.953	(−0.255, 0.240)
Live with one parent							0.141 (0.093)	0.128	(−0.041, 0.323)
Live with two parents							0.080 (0.086)	0.348	(−0.088, 0.249)
Christianity							0.137 (0.073)	0.062	(−0.007, 0.280)
Student							−0.104 (0.103)	0.312	(−0.307, 0.098)
In a vocational training							−0.126 (0.099)	0.201	(−0.320, 0.067)
In a job							−0.238 (0.116)	0.042	(−0.466, −0.009)
Married							−0.114 (0.122)	0.353	(−0.354, 0.127)
Steady relationship							−0.052 (0.083)	0.531	(−0.214, 0.111)
Casual relationship							0.120 (0.105)	0.251	(−0.085, 0.325)

### Multivariable linear regression analysis of intention to use HC among adolescents and young women with HC use experience (*n* = 350) and those without HC use experience (*n* = 853)

Separate regression analyses were carried out for adolescent girls and young women that reported experience with HC use and those that did not, again including only those psychosocial predictors that showed a significant univariate association with intention in each group. Among participants with previous HC use experience, the significant univariate psychosocial correlates of intentions together explained 31% of the variance in intention, with significant unique associations for attitude toward personal HC use and self-efficacy toward access and use of HC. Adding the sociodemographic variables to the regression model did not significantly improve the explained variance in intention to use HC, *Fchange* (12, 214) = 1.70, *p* = 0.066. However, attitude toward personal HC use and self-efficacy toward access and use of HC remained significant. All other measures did not show significant unique associations with intention (see Table 5).

**Table 5:** Multivariable linear regression analysis of intention to use HC among adolescent girls and young women with HC experience (*N* = 350).

	Step 1: multivariable linear regression analysis of HC use intentions among adolescent girls and young women with HC use experience			Step 2: demographic variables included		
	Unstandardized coefficients (SE)	*p*-value	95% C.I (Lower, Upper bound)	Unstandardized coefficient (SE)	*p*-value	95% C.I (Lower bound, Upper bound)
(Constant)	1.095 (0.495)	0.028	(0.120, 2.069)	1.241 (0.565)	0.029	(0.128, 2.355)
Attitude toward HC product	−0.155 (0.127)	0.223	(−0.404, 0.095)	−0.087 (0.128)	0.501	(−0.340, 0.166)
Attitude toward adolescent HC use	0.118 (0.146)	0.422	(−0.171, 0.406)	0.040 (0.149)	0.790	(−0.253, 0.333)
Attitude toward personal HC use	0.439 (0.091)	0.000	(0.259, 0.619)	0.440 (0.092)	0.000	(0.259, 0.621)
Attitude toward discussing HC use with mother	−0.129 (0.071)	0.070	(−0.269, 0.011)	−0.138 (0.071)	0.053	(−0.278, 0.002)
Attitude toward discussing HC use with friends	−0.028 (0.074)	0.706	(−0.175, 0.119)	−0.003 (0.074)	0.965	(−0.150, 0.143)
Attitude toward discussing HC use with partner	0.030 (0.076)	0.692	(−0.120, 0.180)	0.006 (0.076)	0.937	(−0.144, 0.156)
Self-efficacy to access and use HC	0.370 (0.095)	0.000	(0.183, 0.557)	0.361 (0.097)	0.000	(0.170, 0.553)
Self-efficacy to discuss HC use with important others	0.017 (0.091)	0.848	(−0.163, 0.197)	0.050 (0.091)	0.586	(−0.129, 0.228)
Self-regulation skills	0.121 (0.083)	0.148	(−0.043, 0.285)	0.104 (0.082)	0.210	(−0.059, 0.266)
Mother’s normative belief on HC	−0.018 (0.082)	0.829	(−0.179, 0.144)	−0.011 (0.082)	0.896	(−0.171, 0.150)
Friend’s normative belief on HC	0.004 (0.093)	0.963	(−0.180, 0.189)	−0.031 (0.093)	0.736	(−0.215, 0.152)
Partner normative belief on HC	−0.006 (0.079)	0.937	(−0.163, 0.150)	0.001 (0.080)	0.995	(−0.157, 0.158)
Discussing SRH issues with mother	0.049 (0.115)	0.672	(−0.178, 0.275)	0.097 (0.116)	0.401	(−0.130, 0.325)
Severity of consequences of pregnancy	0.080 (0.072)	0.263	(−0.061, 0.221)	0.050 (0.073)	0.492	(−0.093, 0.194)
Pregnancy risk perception	−0.175 (0.082)	0.034	(−0.337, −0.013)	−0.142 (0.083)	0.087	(−0.305, 0.021)
Future ambition	−0.040 (0.102)	0.699	(−0.241, 0.162)	−0.045 (0.103)	0.662	(−0.248, 0.158)
HC knowledge	0.027 (0.051)	0.592	(−0.073, 0.128)	0.038 (0.051)	0.458	(−0.063, 0.138)
Ever been pregnant				−0.209 (0.120)	0.083	(−0.445, 0.028)
Primary/JHS				0.038 (0.209)	0.857	(−0.375, 0.450)
SHS/tertiary				0.044 (0.224)	0.845	(−0.397, 0.484)
Live with one parent				0.259 (0.150)	0.085	(−0.036, 0.553)
Live with two parents				0.298 (0.145)	0.042	(0.011, 0.584)
Christianity				−0.193 (0.134)	0.153	(−0.458, 0.072)
Student				−0.343 (0.184)	0.064	(−0.705, 0.020)
In a vocational training				−0.061 (0.177)	0.729	(−0.410, 0.287)
In a job				−0.171 (0.209)	0.413	(−0.583, 0.241)
Married				0.207 (0.210)	0.325	(−0.206, 0.621)
Steady relationship				0.033 (0.138)	0.810	(−0.239, 0.306)
Casual relationship				0.343 (0.170)	0.045	(0.008, 0.677)

Among participants with no previous HC use experience, adding the significant univariate psychosocial correlates of intentions explained 31.3% of the variance in HC use intention, with significant unique associations for attitude toward personal HC use and self-efficacy toward access and use of HC. As observed in model 2, adding the sociodemographic variables to the regression model did not significantly improve the explained variance in intention to use HC, *Fchange* (12, 449) = 1.82, *p* = 0.042, but the attitude toward personal HC use and self-efficacy toward access and use of HC again remained significant. In addition to this, being a Christian as opposed to participants that affiliate themselves with Islam, Traditional religion or being non-religious positively predicts future HC use (β = 0.233; *p* < 0.01) (see Table 6).

**Table 6:** Multivariable linear regression analysis of intention to use HC among adolescent girls and young women with no HC experience (*N* = 853).

	Step 1: multivariable linear regression analysis of HC use intentions among adolescent girls and young women with no HC use experience			Step 2: demographic variables included		
	Unstandardized coefficients (SE)	*p*-value	95% C.I (Lower, Upper bound)	Unstandardized coefficient (SE)	*p*-value	95% C.I (Lower bound, Upper bound)
(Constant)	0.224 (0.298)	0.451	(−0.361, 0.809)	0.202 (0.328)	0.538	(−0.443, 0.847)
Attitude toward HC product	−0.010 (0.089)	0.912	(−0.184, 0.164)	0.007 (0.088)	0.933	(−0.166, 0.181)
Attitude toward adolescent HC use	0.078 (0.101)	0.442	(−0.120, 0.275)	0.086 (0.100)	0.390	(−0.111, 0.283)
Attitude toward personal HC use	0.229 (0.061)	0.000	(0.110, 0.349)	0.214 (0.061)	0.000	(0.094, 0.333)
Attitude toward discussing HC use with mother	0.027 (0.049)	0.587	(−0.070, 0.124)	0.012 (0.049)	0.816	(−0.086, 0.109)
Attitude toward discussing HC use with friends	−0.043 (0.050)	0.391	(−0.141, 0.055)	−0.046 (0.050)	0.352	(−0.144, 0.052)
Attitude toward discussing HC use with partner	−0.060 (0.050)	0.225	(−0.158, 0.037)	−0.058 (0.050)	0.244	(−0.156, 0.040)
Self-efficacy to access and use HC	0.355 (0.060)	0.000	(0.238, 0.473)	0.340 (0.060)	0.000	(0.222, 0.459)
Self-efficacy to discuss HC use with important others	−0.026 (0.058)	0.659	(−0.140, 0.088)	−0.029 (0.058)	0.613	(−0.144, 0.085)
Self-regulation skills	0.101 (0.056)	0.071	(−0.009, 0.212)	0.087 (0.056)	0.121	(−0.023, 0.197)
Mother’s normative belief on HC	0.012 (0.064)	0.855	(−0.114, 0.138)	0.012 (0.064)	0.856	(−0.114, 0.137)
Friend’s normative belief on HC	−0.036 (0.063)	0.567	(−0.159, 0.087)	−0.024 (0.063)	0.701	(−0.147, 0.099)
Partner normative belief on HC	0.032 (0.060)	0.588	(−0.085, 0.150)	0.037 (0.061)	0.547	(−0.083, 0.156)
Discussing SRH issues with mother	0.129 (0.090)	0.153	(−0.048, 0.306)	0.157 (0.091)	0.084	(−0.021, 0.335)
Severity of consequences of pregnancy	−0.004 (0.047)	0.936	(−0.097, 0.089)	0.005 (0.047)	0.917	(−0.088, 0.098)
Pregnancy risk perception	0.085 (0.048)	0.075	(−0.009, 0.180)	0.090 (0.048)	0.061	(−0.004, 0.184)
Future ambition	−0.053 (0.058)	0.360	(−0.166, 0.060)	−0.054 (0.058)	0.351	(−0.167, 0.059)
HC knowledge	−0.009 (0.033)	0.778	(−0.075, 0.056)	−0.028 (0.034)	0.411	(−0.094, 0.039)
Ever been pregnant				0.149 (0.103)	0.150	(−0.054, 0.352)
Primary/JHS				−0.085 (0.138)	0.537	(−0.357, 0.186)
SHS/tertiary				−0.013 (0.155)	0.933	(−0.318, 0.292)
Live with one parent				0.102 (0.117)	0.386	(−0.129, 0.333)
Live with two parents				−0.007 (0.107)	0.946	(−0.218, 0.204)
Christianity				0.233 (0.089)	0.009	(0.059, 0.407)
Student				0.014 (0.127)	0.915	(−0.236, 0.263)
In a vocational training				−0.099 (0.121)	0.414	(−0.336, 0.139)
In a job				−0.224 (0.142)	0.115	(−0.502, 0.055)
Married				−0.256 (0.153)	0.094	(−0.556, 0.044)
Steady relationship				−0.058 (0.105)	0.583	(−0.264, 0.149)
Casual relationship				−0.020 (0.137)	0.886	(−0.289, 0.250)

In all three models, attitude toward personal HC use and self-efficacy toward access and use of HC showed unique contributions to the prediction of HC use intentions among the adolescent girls and young women (see Tables 4–6).

## Discussion

We explored the social-psychological determinants and background factors that predict HC use intentions among adolescent girls and young women. Among the entire sample of adolescent girls and young women, our results demonstrated the unique contributions of attitude toward personal HC use, self-efficacy toward access and use of HC, and HC use experience, to the explanation of HC use intentions among adolescent girls and young women. Two socio-cognitive factors significantly contributing to the prediction of HC use intentions among the entire sample also predicted HC use intentions in the two sub-samples of adolescent girls and young women based on HC use experience. These were attitude toward personal HC use and self-efficacy toward access and use of HC. In addition to these, being a Christian as opposed to participants that affiliate themselves with Islam, Traditional religion or being non-religious positively predicts future HC use.

All regression models (those including all adolescent girls and young women, and the separate analysis based on HC use, i.e., HC users and non-HC users) showed that attitude toward personal HC use was one of the two most important determinants of HC use intentions in this study. Adolescent girls and young women in our study probably perceive HC use to be very beneficial to them personally in preventing unwanted pregnancy and in turn be able to achieve a future ambition. In a previous qualitative study among adolescent girls with HC use experience in the same area, the girls had positive attitudes toward HC use and they explained how it helped them to remain focused on their schooling or vocational apprenticeship without having to worry about unwanted pregnancy (50). The relationship between positive attitudes toward contraceptive use and intention for future and actual use has been documented before (34, 35, 42). However, the effects of attitudes to personal HC use on HC use intention is rarely reported. Our results imply that adolescent girls will intend to use or actually use HCs if they ascribe personal benefits to their use. This key finding points to the need for interventions that target adolescent girls and young women to help them appreciate the personal gains from using HCs. This could improve their attitudes toward HC use, in order to prevent unwanted pregnancy among them. A positive attitude toward personal HC use can be developed through the application of behavior change interventions, that target attitude such as anticipated regret (46, 54, 55).

Our study further showed that self-efficacy toward access and use of HC was an important predictor of HC use intentions in the entire sample of respondents and in the sub-samples based on HC experience. Literature provides evidence in support of our findings (33, 34, 44, 45, 56), and so do our previous qualitative findings among adolescent girls with and without HC use experience in the same study area (41, 50). Among adolescents with HC experience, probably they are strong willed and resilient and are able to access their HC methods without barriers. This assertion is based on findings from our previous qualitative studies (41, 50). The girls using HCs in that study compared to those who had stopped using theirs showed much stronger self-efficacy to access HCs from the various health outlets and also used them consistently. They were resilient and not too worried about meeting other people in those places who might tag them as bad girls and misjudge them compared to the girls not using HC who were shy, afraid and embarrassed to seek for HCs from various source (41, 50). The findings from these studies indicate that all other things being equal, self-efficacy to access and to use HC is an imperative skill to possess by adolescent girls and young women for successful HC use. The results call for tailor-made interventions that will target and provide adolescent girls and young women with the needed skills to be able to access and use HCs if need be. Interventions like “Health-E You” used an interactive app, to provide individually tailored health education on contraceptives, their use and decision-making support for Latina adolescents. This significantly improved their self-efficacy, HC use decision-making skills, and uptake of contraceptives among them (57). The use of mobile phones is common among Ghanaian adolescents and it will be worth trying out such interventions for them.

HC experience was found to positively predict HC use intentions in this study. Probably most study participants have benefited from using HCs, which motivates their intentions for future use and this finding situates well in the literature (20, 41, 58, 59). Also, satisfaction with past contraceptive method use is associated with intention for future HC use (59). Therefore, adolescent girls and young women who were satisfied with the methods they used in the past, are likely to continue their use in future. On the other hand, health concerns and fear associated with unpleasant effects from HCs account for why many women opt for non-HC use and also for method discontinuation (20, 41, 58), especially in Ghana (20, 47). Hence, for adolescent girls to continue using their HCs, comprehensive education on HCs about how they work, their benefits and correcting possible misperceptions would be very beneficial among adolescents for their future HC use decision-making.

Among respondents with no HC experience, being a Christian as opposed to being a Muslim, Traditional worshiper or having no religion positively predicts future HC use. This finding is likely by chance.

Religious groupings in Ghana regard sex before marriage as sinful therefore, premarital sex and the use of HCs may not be condoned by adolescent girls and young women who endorse these religious norms. The influence of religion and religious beliefs in adolescent HC use is shown in the literature (34, 58, 60, 61). The role of religious normative beliefs is crucial in adolescent HC use decision-making (50). Therefore, the design of any intervention among adolescent girls aimed at improving their sexual and reproductive health and rights particularly the use of HC, should consider the influence of religious beliefs and make the necessary contextually appropriate provisions for that (62).

The models used to identify the determinants of HC use intentions in this study explained between 31 to 43% of the variance in HC use intentions among the different sub-samples of adolescent girls and young women studied and this is comparable to other study findings (35, 63). However, there is still around 58% of variance in HC use intentions to be explained. In addition to socio-cognitive factors, previous empirical studies have also shown that structural factors such as poverty, cultural norms, healthcare access, and gender inequality are important factors in contraceptive use intentions and actual use (11, 64). These factors among others could account for the rest of the unexplained variance in HC use intentions in this study. It would be important to assess the associations of HC use intentions with some of these endpoints in future.

Our current study findings should be interpreted with some caution due to a few limitations. We used a survey that was administered to the participants using face-to-face interviewing, thus a bit less privacy for them. This could produce social desirability bias. We tried to minimize this limitation of probable data misreporting by fully assuring participants of anonymity and confidentiality. Also, using cross-sectional survey made it impossible to make casual inferences in this study but our results point toward important endpoints for future research in that regard and also show appropriate gaps for future interventions.

In conclusion, our results demonstrate that different groups of adolescent girls need different interventions, focusing on different determinants for improved HC use intentions and actual HC use. It is not one size fit all. However, overall, attitude toward personal HC use and self-efficacy to access and use HC were crucial for positive HC use intentions. Through comprehensive sexuality education including information provision, persuasion, modeling, and guided practice, adolescents’ attitude to personal HC use and their self-efficacy to access and to use HCs can be improved for successful HC use among different adolescent groups in our setting and other similar settings.

## Data availability statement

Data supporting the conclusions of this article will be made available upon request in accordance with the Kintampo Health Research Centre data sharing policy.

## Ethics statement

The studies involving human participants were reviewed and approved by Kintampo Health Research Centre Institutional Ethics Committee (Federal Wide Assurance number 00011103) in Ghana and the Ethical Review Committee, Psychology, and Neuroscience at Maastricht University (ECP_04_09_2012_S23) in the Netherlands. Written informed consent to participate in this study was provided by the participants’ legal guardian/next of kin.

## Author contributions

EB-K and FM conceived the idea for the work. EB-K, RR, and FM developed the questionnaire. RR and EB-K analyzed and interpreted the data. KA and SO-A supported study planning and implementation. EB-K wrote the first draft of the manuscript with support from RR. FM, SO-A, and KA were reviewed the subsequent versions of the manuscript. All authors approved of the final version submitted.

## Funding

This work was funded by the Kintampo Health Research Centre.

## Conflict of interest

The authors declare that the research was conducted in the absence of any commercial or financial relationships that could be construed as a potential conflict of interest.

## Publisher’s note

All claims expressed in this article are solely those of the authors and do not necessarily represent those of their affiliated organizations, or those of the publisher, the editors and the reviewers. Any product that may be evaluated in this article, or claim that may be made by its manufacturer, is not guaranteed or endorsed by the publisher.
